# Supplementary material for: Are carbon emissions trading and green financial instruments synergistic? -Comprehensive quantitative research based on content analysis
Source: PLoS One. 2024 Mar 7;19(3):e0298601. doi: 10.1371/journal.pone.0298601 (PMC10919601; doi:10.1371/journal.pone.0298601)
Supplement: S1 Data — (ZIP) [file pone.0298601.s001.zip › Data/地级市Z_F工作报告.zip!/╕ú╜¿/╚¬╓▌╩╨/└┤╫╘╣1⁄2╓┌║┼í╛┬φ┐╦╩2╛▌═°í┐_2009.docx]

各位代表：

现在，我代表泉州市人民政府向大会作工作报告，请予审议，并请政协委员和其他列席人员提出意见。

一、2008年政府工作回顾

过去一年，是改革开放30周年和我国发展进程中很不寻常、很不平凡的一年，也是我市具有特殊意义的一年。在市委领导下，我市各级政府各部门认真贯彻落实党的十七大和十七届三中全会精神，以邓小平理论和“三个代表”重要思想为指导，深入学习实践科学发展观，按照省委、省政府海西战略部署和"科学发展、四求先行"要求，有为应对国际金融危机带来的困难和挑战，全市实现生产总值2700亿元（预计，下同），增长14%；财政总收入263.6亿元，增长17.1%，其中，一般预算收入137.17亿元，增长19.7%，基本实现了市十四届人大三次会议确定的经济社会发展目标。成功举办了第六届全国农民运动会，这是新中国成立以来我省承办的规模最大、规格最高、成效最好的大型体育盛会，有力地促进了泉州各项工作，提升了泉州的形象，扩大了泉州的影响，赢得了广泛赞誉。同时，我市还被列为全国18个改革开放典型地区之一，荣获中国十佳和谐可持续发展城市、中国品牌之都、建设创新型国家十强市、中国城市综合创新力50强、创建全国文明城市工作先进城市等称号。

一年来，我们的主要工作和成效是：

直面困难积极有为应对，力促经济保持平稳较快增长。针对严峻国际经济形势影响，按照国家宏观调控政策调整的要求，深入各县（市、区）、泉州开发区和基层、企业调研，掌握情况，与企业界人士分析困难、研究措施、共商对策。按照“稳住大盘、固本培源”工作思路，强化阶段性预测预警，逐季召开全市经济运行分析会，逐月跟踪经济发展情况，加强生产要素保障和经济运行调度。根据形势变化和企业需求，5月份出台《关于加强中小企业信用担保体系建设的实施意见》，6月份出台《关于加强企业上市服务协调工作的通知》，9月份下发《关于做好第四季度工作和及早谋划2009年工作的通知》，11月份研究《关于扶持规模以下成长型工业企业的若干意见（试行）》，12月份出台《关于贯彻落实中央和省进一步扩大内需促进经济增长的实施意见》等文件。召开千名企业家大会，鼓励企业增强信心，优化管理，化“危”为“机”，战胜困难。发挥行业协会作用，推动企业加强互助合作。支持帮助纺织、服装等行业争取提高出口退税率等优惠政策，增加退(免)税7.23亿元，全年办理出口货物退（免）税达39.6亿元。推动政银企携手，召开全市金融工作座谈会，成立全省首家小额贷款公司，推荐94家企业获得银行授信，全市新增银行贷款225亿元；新增上市企业5家，融资30.8亿元。加强用地监管和保障，抓好房地产用地调控，出台居民购房支持政策。认真落实外来员工“十项待遇”，努力稳定企业员工队伍。各县（市、区）、泉州开发区和市直各有关部门也结合实际，强化服务，出台了一系列有利于企业发展的政策措施，全市经济大盘基本保持稳定。

认真落实强农惠农政策，“三农”工作取得新成效。加大“三农”投入，全市支农支出增长18.5%。落实粮食订单收购和最低收购价政策，兑付粮食、生猪、渔业等生产补贴资金5.07亿元。抓好178个省市县新农村建设试点示范村和83个扶贫工作重点帮扶村，加强镇村规划建设，基本完成新农村建设“十件实事”。大力发展现代农业，新增国家级、省级农业产业化龙头企业2家和6家，农业“三品”认证38个，中国名牌农产品2个。组织实施《海峡两岸（泉州）农业合作试验区发展规划》，成功举办首届海峡两岸（泉州）农产品采购订货会及泉州龙眼展示会。加强农业基础设施建设，完成外走马埭围垦海堤工程、晋江下游防洪岸线（二期）金塔段、山美灌区、南渠改造整治项目及“六千”水利工程和深沪、祥芝、崇武三大国家级中心渔港年度建设任务。扎实做好防汛工作，战胜“海鸥”、“凤凰”等台风暴雨袭击。加强重大动植物疫病防控工作。逐步推进土地承包经营权流转和林业投融资体制改革，农村“七大员”队伍建设进一步加强。

大力推进发展方式转变，产业结构进一步调整优化。强化自主创新，组织实施国家、省、市级科技项目191项，新增国家级企业技术中心2家、示范生产力促进中心1个，参与国际、国家、行业和地方标准制（修）订企业分别达2家、23家、10家和12家。新增中国驰名商标（国家工商总局认定）3件、中国行业标志性品牌8个。中国纺织工业协会检测中心落户泉州。加强产学研合作，“6·18”项目成果交易会成功对接项目665个。继续扶持行业龙头企业发展，全市规模以上工业产值占工业总产值比重达84.8%，新增产值超亿元企业186家。出台实施《关于加快发展服务业的实施意见》，第三产业增加值增长14.7%；社会消费品零售额906亿元，增长20%；旅游总收入198亿元，增长15.1%。转变外贸发展方式，实现出口额（海关口径）58亿美元、出口交货值119亿美元，分别增长16%和11%。实际利用外资（验资口径）17亿美元，增长56.7%；新批投资总额（含增资）超千万美元项目83个。深化泉台经贸合作，和谐光电、创辉光伏等一批重点台资项目顺利推进。

深入实施项目带动战略，城市发展空间进一步拓展。147个年度在建重点项目完成投资225亿元（不含福建炼油乙烯一体化），占年度投资计划101%。泉州晋江大桥、泉三高速公路泉州至永春段等64个项目建成或部分建成，福厦铁路泉州段、晋江燃气电厂、泉州船厂修船等一批项目实现年度建设目标。中心市区城建项目完成投资48亿元。《泉州城市总体规划（修编）》报省政府审批，东海、洛秀、北峰丰州和城东分规控规及专项规划基本编制完成。海峡体育中心、泉州迎宾馆投入使用，（下转第二版）上接第一版）金浦水厂（一期）、泉州市液化天然气利用工程顺利实施。道路交通综合整治工作得到加强，市区4座人行天桥建设、106条市政道路路面修复和13条主干道路面加铺沥青混凝土改造完成，市区主干道市容市貌及景观整治取得成效。城东、东海、桥南三大片区建设步伐加快，中心市区建成区面积增至91平方公里，全市城镇化率达55%。亿吨大港建设扎实推进，港口完成投资10.7亿元。肖厝、斗尾等重点港区和肖厝、秀涂港后区控制性详规编制完成，惠安青兰山30万吨级原油专用码头、泉州湾深水航道、石湖4#泊位、围头防波堤等一批港口项目基本建成。围头、深沪港区通过国家口岸开放验收。泉州中心港区、肖厝港区被列为两岸海运直航港口，泉州港实现对澎湖和台湾本岛海上双向货运直航，泉金航线客货运量分别增长38%和93%。全市港口货物和集装箱吞吐量达7220万吨和119万标箱，分别增长16.2%和16.8%。

不断加大社会建设力度，文教体卫事业发展取得新成绩。全市投入教育经费41.43亿元，增长18.1%，其中落实“两免一补”经费3.03亿元，投入近1亿元改造中小学校危房7.77万平方米、改造扩建新建校舍10.05万平方米。中小学校教育资源进一步整合优化，洛江区通过省“双高普九”验收，高中阶段教育毛入学率达75.24%。继续开展赴省外招才引智工作，博士后工作站新进站博士15人，引进各类专业技术人才3000多人。加强卫生工作，市第一医院新院急诊部建成投入使用，泉州医疗中心（一期）传染病院完工，市人民医院新院续建工程完成建设，手足口病、霍乱等疫病得到有效防控，20个重点乡镇卫生院和10个社区卫生服务中心配套建设顺利完成。继续推进闽南文化生态保护工作，新入选国家级非物质文化遗产项目17个，泉州南音列入国家申报“世界非物质文化遗产代表作”正选项目。泉州提线木偶参与北京奥运会开幕式演出。我市运动员在北京残奥会、第六届全国农运会上分别获得1金1铜和17金14银4铜的好成绩。全市人口出生率11.12‰，政策符合率95.15%，保持低生育水平。加强防震减灾体系建设，开展建筑物抗震安全评估，全市改造危旧房93万平方米。启动第二轮修志工作。组织防灾防空应急演练。加强国防后备力量建设和民兵预备役工作，支持部队现代化建设，深入开展双拥共建活动。做好支援四川灾区及对口支援彭州恢复重建工作，全市累计向四川地震灾区捐赠款物4.19亿元。

致力保障和改善民生，社会保持和谐稳定。全市城镇居民人均可支配收入和农民人均纯收入达20270元和7970元，分别增长12%和10%。全市居民消费价格上涨4.0%，涨幅控制在预期目标以内。社保覆盖面继续扩大，参加城镇职工基本养老、医疗、失业、工伤、生育保险人数分别增至66.5万、57万、46.5万、49.8万和44.09万人。参加职工医疗互助42万人。启动城镇居民基本医疗保险，参加人数67万人。提高新型农村合作医疗标准，全市参合人数增至495.17万人。享受城乡低保12.09万人。慈善事业进一步发展，新增签约认捐善款6.58亿元。全市企业劳动合同覆盖率95%，职工劳动合同签订率88%。城镇新增就业15.5万人，城镇登记失业率1.2%。完成中侨公司等一批国有企业和5家城镇集体企业改制及职工安置工作。推进保障性住房建设，全市在建、拟建保障性住房项目27个，完成建筑面积33.19万平方米，其中廉租住房550套、2.75万平方米。加强产品质量和食品药品安全监管，认真开展乳制品质量安全专项治理。做好“侨居造福工程”。推进“五五”普法。深化“平安泉州”创建活动，加强社会治安综合治理，做好信访、人民调解、社区矫正工作，群众对社会治安满意率提高1.29个百分点。启动落实企事业单位安全生产主体责任“三年行动”，开展“安康杯”竞赛活动，安全生产综合指数同比实现“三降一升”。

不断强化节能减排工作，生态文明建设扎实推进。组织实施循环经济示范项目20个，完成二氧化硫和化学需氧量结构调整项目270个，预计可实现全年的节能减排目标。加强饮用水源保护，完成“两江”上游水资源保护项目26个，晋江干流上游水质保持在Ⅱ类标准。全市投入近海水域环境污染整治资金10.2亿元，完成项目77个。在全省率先实现所有县（市、区）建成一座以上污水处理厂，全面开征垃圾处理费，中心市区污水处理率和垃圾无害化处理率分别达83%和99%。规范国有土地出让，加强土地节约集约利用，全市共清理闲置土地174宗，面积253.8公顷，基本完成中心市区土地出让金清缴工作。加强生态公益林保护，植树造林13.8万亩。

依法行政工作得到加强，政府公共服务水平不断提高。认真执行市十三届人大七次会议和市十四届人大一次会议议案决议。深入贯彻《监督法》，完成“十一五”规划实施情况中期评估工作。积极配合市人大开展执法检查，支持和保障人大代表、政协委员依法开展工作。认真办理人大代表建议386件、政协委员提案425件，办结率均达100%。规范行政处罚自由裁量权试点工作取得较好成效。晋江、石狮、南安三个县级市率先获省政府批准开展相对集中行政处罚权工作。第八轮行政审批制度改革扎实推进，全程式网上审批拓展深化，行政服务中心运作进一步规范。贯彻实施《政府信息公开条例》，大力推进政务公开工作。规范公务员和中小学、幼儿园教师津贴补贴。稳步推进事业单位改革。开展市场中介组织专项整治。加强机关效能监察和绩效评估。持续深化反腐倡廉工作，加大案件查处力度，处理违法违纪政府机关工作人员93人，挽回经济损失423.21万元。完成省下达的30项为民办实事项目和市委、市政府11件27项为民办实事项目。

各位代表，面对复杂多变和严峻的经济形势，面对经济社会发展中的诸多矛盾和问题，我们深感成绩来之不易。这是省委、省政府和市委正确领导的结果；是全市干部群众团结一心、奋力拼搏的结果；是市人大、市政协有效支持监督，各民主党派、工商联、无党派人士、各人民团体和离退休老同志关心支持，驻泉省部属单位、驻泉部队、武警官兵，企业界人士、来泉务工人员及广大港澳台同胞、海外侨胞、海内外朋友同心协力、共同奋斗的结果。在此，我代表市人民政府向所有关心支持泉州发展的各级领导、各界朋友和海内外人士致以崇高的敬意！对人大代表、政协委员和全市人民给予政府的信任和大力支持表示衷心的感谢！

在看到成绩的同时，我们也清醒地认识到，我市经济社会发展还存在不少困难和问题。主要是：受国际金融危机不断扩散和国内经济运行困难增加的影响，对外经贸发展呈下滑趋势，部分企业和行业经营困难；产业结构有待优化，现代服务业、高新技术产业发展不快；农业增产、农民增收和农村劳动力转移就业难度加大，农村社会事业发展相对滞后；城市化进程滞后于工业化发展，中心市区辐射和带动能力不强，交通间歇性拥堵现象仍然存在；土地、水等资源紧缺更加突出，节能减排形势仍较严峻；社会保障工作任务艰巨，社会管理和公共服务水平有待提高；稳定低生育水平任务繁重，政策外生育和性别比失衡问题仍未得到根本解决；应对自然灾害和突发性公共事件的工作机制有待进一步健全，食品药品安全监管工作亟待加强，安全生产形势仍较严峻；基层矛盾纠纷时有发生，社会治安防控体系仍需完善；个别行政部门的权力运行不够规范，个别公务人员行政不作为、乱作为现象仍有发生；反腐倡廉工作还需进一步加强。此外，2008年市《政府工作报告》提出的248项任务有2项尚未完成，17个年度预期指标有3个未达到预期要求。上述问题需要我们进一步采取措施，认真加以解决。

二、2009年政府工作的指导思想和预期目标

2009年是新中国成立60周年，也可能是我市经济发展最为困难但又蕴含重要机遇的一年。从国际环境看，世界金融危机持续加深，全球经济增长明显放缓，保持我市经济持续增长的压力进一步加大。从国内环境看，国家把保持经济平稳较快发展放在首要位置，实行积极的财政政策和适度宽松的货币政策；两岸关系出现重大积极变化，两岸“三通”正式实现，泉台交流合作正处于新的起点。从我市实际看，经过改革开放30年的努力，泉州新一轮发展的基础日趋厚实，企业具备较强的抵御风险能力，我市仍处于发展的重要战略机遇期。我们坚信，在市委领导下，泉州一定能够继续坚持改革开放，妥善应对危机和挑战，把握机遇，奋力先行，突破风险，迎难而上，保持经济平稳较快增长。

2009年政府工作的指导思想是：全面贯彻党的十七大、十七届三中全会精神，以邓小平理论和“三个代表”重要思想为指导，深入学习实践科学发展观，认真贯彻省委八届五次全会和市委十届四次全会精神，牢牢把握“科学发展、四求先行”实践主题，围绕建设海峡西岸经济区现代化工贸港口城市目标，着力推进产业发展、港口发展、城市发展，坚持稳住大盘、固本培源、项目带动、奋力发展，加快转变经济发展方式，统筹推进农村改革发展，致力保障和改善民生，继续在推进海西“两个先行区”建设中走前列。

2009年我市经济社会发展主要预期目标为：地区生产总值增长11%；财政总收入增长15%，其中一般预算收入增长14%；规模以上工业产值增长17%；工业增加值增长11.5%；第三产业增加值增长12%；全社会固定资产投资增长20%；外贸出口增长10%；实际利用外资（验资口径）持平；城镇登记失业率控制在2%以内；社会消费品零售额增长14%；居民消费价格总水平涨幅控制在3.5%；人口自然增长率控制在8.7‰以内；城镇居民人均可支配收入增长8%；农民人均纯收入增长8%；万元生产总值能耗下降3.2%，二氧化硫排放量下降4.2%，化学需氧量排放量减少1.1%。

三、稳住大盘，固本培源，推动经济持续较快发展

保持经济持续较快增长是今年我市经济工作的首要目标和任务。要有效落实国家、省、市扩大内需和扶持企业发展的一系列政策措施，竭尽全力“保企业、保市场、保要素供给”，全力稳住工业经济大盘；坚持把保增长、扩内需、调结构有机结合起来，大力推进“项目建设年”活动，集中加快建设和启动一批促进结构优化、完善基础设施、支持“三农”、改善民生、节能环保等方面的重大工程，加大新兴产业及高科技产业培育力度，加快发展方式转变和经济结构调整进程。

推进现代产业发展。强化项目带动。全年计划实施167个在建重点项目，实现年度投资300亿元以上。五大传统产业要突出抓好79个产业升级项目，实现年度投资36亿元以上；五大新兴产业及高新技术产业要重点推进福建炼油乙烯一体化、中化重油深加工、石狮PTA、泉州船厂、钧石能源、三晶阳光、金保利电子、泉州微软技术中心、修正药业、太古飞机维修制造等51个项目建设，实现年度投资109.8亿元，尽快形成规模生产力。强化产业协作。抓好18个产业基础设施配套项目，完成年度投资15.3亿元。发挥行业协会作用，支持龙头企业开展兼并重组、改制上市、品牌营销，引导中小企业与龙头企业建立互补配套、合作共赢机制。抓好100家重点工业企业和50家省级新增长点企业，扶持一批效益好、潜力大、符合产业导向的规模以上企业发展。继续实施规模以下企业成长工程，加大对中小企业的扶持力度。强化要素保障。认真落实国务院关于当前金融促进经济发展的30条政策措施，鼓励金融机构加大信贷投放力度，支持重点项目建设和中小企业发展。发挥财政资金的杠杆作用，用好增值税转型改革政策。深化投融资体制改革，探索构建国企投融资平台。科学、依法安排用地指标，启动外走马埭围垦工程垦区建设，完善耕地异地占补平衡机制，提高土地节约集约利用水平。强化自主创新。认真实施4个市级重大科技项目和200个科技计划项目，市、县两级财政投入科技经费3.43亿元，新增市级以上研发机构20家。实施知识产权和标准战略。充分发挥“6·18”项目成果交易会平台作用，继续抓好签约项目落实。大力推进信息化与工业化融合，加快运用信息技术改造企业步伐。强化节能减排。抓好六大节能领域、十大节能工程、20个循环经济示范项目和百家重点耗能企业的节能工作。继续推进建陶行业水煤气综合整治和造纸、制革行业污染治理，加快石粉利用等项目开发。大力发展现代服务业。强化消费拉动，积极培育城市商业圈，不断扩大农村消费市场，促进消费升级。加快金融保险、现代物流、科技信息、工业设计、文化创意、商务会展、社区服务等服务业发展。抓好海峡西岸国际采购与区域物流中心等30个服务业项目，实现年度投资17.7亿元。引导和促进房地产业健康发展。加大旅游资源整合提升和推介力度，大力发展文化旅游、滨海旅游、生态旅游，着力抓好南少林寺片区改造、泉州木偶剧院等文化旅游项目建设，培育旅游新增长点。加强招商选资工作。发挥泉州出口加工区、晋江和南安装备制造业基地及各地工业园区的载体作用，强化跟踪服务，督促已签约项目尽快建成投产。重视发挥海内外泉商和127家异地商会的作用，实施“回归创业工程”，携手共建美好家园。努力保持出口增长。用好出口退税、外贸发展专项资金、出口信用保险等政策措施。建好纺织服装、鞋业、石材出口基地，推进国际电子商务应用平台综合试点。优化出口市场结构，提高自主品牌出口比重，鼓励企业“走出去”设立营销网络，开发新市场，有为应对国际贸易壁垒。推动泉台经贸深度合作。积极扩大“三通”效应，认真实施《泉台产业对接专项规划》，大力推进洛秀组团、泉港台湾石化专区等载体建设，承接台湾电子、机械、石化、物流等产业转移，推进台资企业生产性原料及设备零部件配送物流中心建设，打造台资聚集区。继续跟踪台商投资区申报工作。推进闽台农产品交易市场建设，做大对台小额贸易，办好海峡两岸（泉州）农产品采购订货会，努力打造两岸农产品贸易平台。同时，要不断提高泉州与香港、澳门和东盟的经贸合作水平；加强侨务工作，密切与海外侨胞侨亲的联络联谊，做好华裔新生代和华文教育工作，继续推进华侨农场的改革发展，关心贫困归侨、侨眷生产生活。

推进港口建设发展。积极争取《泉州港总体规划》和重点港区控制性详规获批。加大港口投资力度。抓好石湖5#泊位、泰山石化码头、中化库区码头、惠安小岞万吨级散杂货码头等项目建设。基本完成围头湾通海航道工程，完善新建码头的装卸设备和堆场。启动湄洲湾南岸疏港铁路和海铁联运项目。继续推进石湖港石材物流园区建设。全年完成港口及疏港通道建设投资25亿元。用心培育航运市场。认真落实港口生产优惠政策，扶持发展航运、港口物流、集装箱联运、船货代理等中介服务机构，吸引本外地货源从泉州港进出，努力提高港口货物和集装箱吞吐量。不断扩大港口业务。巩固内贸集装箱区域枢纽港地位。促请中远集团加大投资，适时增加开通泉州直航台湾及波斯湾集装箱班轮等航线。抓紧做好与台湾港航企业的业务对接工作，扩大对台海上货运直航成果，深化与金门、澎湖海上客货直航合作，把泉州港打造成为两岸直航的重要通道。完善大通关机制。深化港口管理体制改革，推进电子口岸信息平台建设，实行更加便捷的查验通关服务。深入开展打击走私活动。提升防范和应急处置海上交通事故、污染事故的能力。

推进城市空间拓展。进一步完善城市大交通体系。完成铁路、高速公路建设、机场扩建年度投资56.7亿元。确保泉三高速公路泉州段全线通车、福厦铁路泉州段建成调试，泉三高速公路南惠支线、泉厦和福泉高速公路扩建工程泉州段、沈海高速公路复线南安（金淘）至厦门泉州段实现年度建设目标。做好泉州环城高速公路晋江至石狮段、南安至石井段、泉州湾跨海通道工程和海西高速公路网莆永线永春至永定泉州段、永春至莆田泉州段等项目的前期工作。启动泉州晋江机场扩建项目。进一步撑大城市框架。强化泉州湾都市区规划协调，推进各区域专项规划，加强对县城、镇村规划的指导。有序开发城东、东海、江南、北峰、洛秀及晋江下游两岸等区域，推进滨江环路及配套工程、城东市政道路、东海市政道路、东海隧道工程、泉州火车站综合客运交通枢纽、泉州火车站站前大道和站前大桥等城市交通路网建设。进一步提升城市综合服务功能。出台加快总部经济发展的政策措施，规划建设企业总部经济集聚区，强化城市商务、文化服务功能。完善和落实现有人才政策，发挥博士后工作站的作用，积极引进符合我市发展需要的高层次人才、留学人员和紧缺人才，强化人才集聚效应。继续推进南埔电厂（二期）、石狮鸿山热电厂、晋江燃气电厂、500千伏大园至晋江变电站线路、泉州电网改造工程、泉州液化天然气利用工程等项目建设，推动晋江华电、惠安福煤电厂等前期工作，强化城市能源保障。进一步改善城市人居环境。认真执行市十三届人大七次会议议案决议，继续抓好近海水域环境污染和晋江、洛阳江流域及城市内沟河环境综合整治。加强饮用水源保护与管理，完善“两江”上下游生态补偿机制。加大城市污水处理厂收集管网建设力度，强化日常监管，提高城市生活污水处理厂运行效益。抓好重点区域乡镇之间联合筹建污水处理厂的试点工作。推进垃圾收集、转运、处理体系建设。实施中心市区金浦水厂（一期）、城市供水管网改造等工程，规划建设一批绿地、停车场。继续深入制止“两违”。完善城市社区便民服务设施，提高社区管理水平。认真执行市十四届人大一次会议议案决议，逐步建立城市道路交通综合整治长效管理机制。

四、大力推进农村改革发展，全面加强以改善民生为重点的社会建设

切实把“三农”放在重中之重位置。认真贯彻党的十七届三中全会精神和省委、市委推进农村改革发展的实施意见，创新体制机制，提升“三农”工作实效。大力发展现代农业。加快特色农业基地建设，积极扶持发展农业产业化龙头企业，重点抓好6类14种特色农产品的基地化、集约化、标准化、信息化和品牌化建设。加强粮食生产和储备，提高粮食最低收购价格，稳定240万亩粮食播种面积。抓好重大动植物疫病和外来有害生物防控。整顿规范农资市场。扩大泉台农业合作领域，深入实施《海峡两岸（泉州）农业合作试验区规划》。落实惠农利农政策。重点抓好农业补贴、农业投入保障、农产品价格保护、农业生态补偿制度等政策落实。市本级今年安排支农支出2.16亿元，增长11.7%，高于市本级可支配财力增幅1个百分点。各县（市、区）支农支出也要达到法定增长幅度要求。继续深化农村改革。严格农村土地管理，逐步规范农村土地承包经营权流转行为。完善征地补偿标准，确保土地出让金收入按政策规定投入农业。推进“三农”担保方式改革创新，扶持发展农民专业合作社、专业大户、家庭农场等规模经营主体。实施农村劳动力转移就业培训“阳光工程”。开展新型“三农”服务体系试点建设，完善农村“七大员”管理制度。重视农业基础设施建设。加强农村公路、电网建设管护，完成海堤、水库和山围塘除险加固年度任务。推进晋江下游防洪岸线金潘段整治、山美水库大坝防渗加固、桃源水库扩蓄等项目建设，开展碧坑水库前期工作。改善农村生产生活条件。推广晋江“百企联百村”等经验，抓好16个省级、20个市级和一批县级新农村试点示范村建设，不断提升原有示范村建设水平。继续实施“万村千乡市场工程”。深入开展农村“家园清洁行动”。加强水土保持工作。推进沿海防护林和绿色通道两项重点生态工程建设。帮扶10个省级、20个市级扶贫开发重点村，扶持老少边岛地区发展。推进农村社区建设试点工作。实施“造福工程”，完成搬迁6000人任务。

统筹城乡社会事业发展。协调发展各类教育。落实义务教育免费和补助政策，继续推进中小学校布局调整，扶持15所农村寄宿制学校配套建设，加快中小学危房改造步伐。全面推进素质教育，推动泉港区通过省“双高普九”评估验收。加快发展普通高中教育和职业教育。科学发展高等教育。规范发展民办教育。重视学前教育、特殊教育。完善终身教育体系，建设学习型社会。大力发展卫生事业。积极稳妥推进医药卫生体制改革，加快构建覆盖城乡的新型公共医疗卫生服务体系。推进市第一医院新院、福建医大附属第二医院新院和泉州医疗中心建设，抓好一批城市社区卫生服务中心和重点乡镇卫生院标准化建设，扶持妇幼卫生事业发展。加强重点传染病防控工作，增强应对突发公共卫生事件能力。整顿规范医疗服务和药品流通市场。不断提升文化软实力。抓好争创第三届全国文明城市工作，广泛开展文明村镇、文明集市、文明户、志愿服务等群众性精神文明创建活动。推进文化产业发展。出台《闽南文化生态（泉州）保护实验区实施意见》，组织13个文化生态保护示范点（示范园区）建设。继续推进“海丝”申报“世遗”和泉州南音申报“世界非物质文化遗产代表作”。完善覆盖城乡的公共文化服务体系，建设改造18个乡镇综合文化站、200个村文化活动室和200家农家书屋。开展“万千百十”文化惠民工程。举办首届“闽南文化节”，组织好对台文化交流系列活动。加强人口计生工作。坚持计生基本国策，推进优生优育。强化出生人口性别比失衡问题综合治理，进一步提升流动人口计生服务管理水平，完善和规范人口计生利益导向机制，继续稳定低生育水平。巩固发展军政军民团结。切实加强国防后备力量建设和双拥支前工作，以评选省新一轮“双拥模范城”为契机，认真落实省、市双拥工作若干意见及实施办法，抓好军转干部安置、随军家属就业和优抚安置等工作。同时，要深入实施《泉州市妇女儿童发展纲要》，依法保障妇女和未成年人权益；统筹抓好民族宗教、广播电视、新闻出版、外事、体育、地方志和《泉州文库》编修、档案管理、人民防空、老区建设、老龄、残疾人、关心下一代等工作。

加强劳动和社会保障工作。实施积极的就业政策。培训各类劳动力25万人，新增城镇就业10万人。完善创业促进就业服务体系，支持自主创业、自谋职业，建成市劳动力中心市场，推进失业动态重点监测报告制度试点。做好高校毕业生就业指导服务及零就业家庭人员就业工作。妥善处理劳动关系。健全劳动争议处理联动协调机制，探索建立农民工工资支付保证金制度，建立市直及县（市、区）欠薪保障调节应急基金制度，确保企业用工合法有序和职工合法权益得到维护。完善社会保障体系。进一步健全“五险统征”机制，职工参加养老、医疗、失业、工伤、生育保险人数扩至71.5万、62.5万、47万、55万和46万人以上。大力推进城镇居民基本医疗保险，力争参保人数达100万人以上。加快新型农村合作医疗信息系统建设，力争全市农民参合率达90%以上。继续推进来泉务工人员、农民工住院医疗保险和职工医疗互助活动，探索建立农民工养老保险、被征地人员养老保险制度。继续提高企业退休人员养老保险待遇。加快构建覆盖城乡的新型社会救助体系，继续发展社会福利和慈善事业，完善城乡居民最低生活保障制度。健全廉租住房制度，推进保障性住房建设。强化防震减灾及抗震设防工作。完成防灾减灾投资5.3亿元。逐步推进石结构房和危旧房改造。建设市级救灾物资储备库。继续抓好支援彭州灾区恢复重建和援宁、援疆、援藏工作。

全力维护社会和谐稳定。推进“五五”普法，组织实施新一轮依法治市规划。完善社会矛盾纠纷排查调处机制，深化“十百千万”人民调解示范活动，巩固社区矫正试点成果。推进基层依法治理，完善村（居）民自治制度。拓展“平安泉州”创建活动内涵，加强流动人口、重点人群、社会组织的服务管理。总结推广“综治进民企”、“十厂十店联防”等经验，有效防范和依法打击各种违法犯罪活动，促进社会治安防控工作社会化，争创全国“长安杯”。加强诚信建设，继续严厉打击“假冒伪劣”产品。高度防范并妥善处理因企业倒闭、逃废债务而引发的群体性事件。进一步强化安全生产监督管理，扎实有序推进企事业单位安全生产主体责任“三年行动”，有效减少安全生产事故总量，坚决杜绝重特大安全生产事故发生，促进安全生产形势持续好转。加强食品药品安全防范与监督管理，深入治理“餐桌污染”，建设“食品放心工程”。落实《突发事件应对法》，完善突发公共事件应急管理机制。

五、强化政府服务保障，提高领导科学发展的能力水平

新形势、新任务对政府工作提出了新的更高的要求。各级政府各部门要增强忧患意识，保持清醒头脑，充分估计可能遇到的困难和风险，以深入学习实践科学发展观活动为契机，切实加强自身建设，更加有效作为，更加科学运作，更加主动服务，以实实在在的工作成效增强执行力、公信力，为推进科学发展提供强有力的保障。

坚持科学、民主、依法。深入贯彻《监督法》，认真执行人大及其常委会决议和决定，自觉接受人大及其常委会的法律监督和工作监督，认真办理人大代表建议。重视和支持人民政协履行政治协商、民主监督、参政议政职能，认真办理政协委员提案。重视听取各民主党派、工商联和无党派人士的意见，发挥工会、共青团、妇联等人民团体的作用。坚持重大事项集体研究决定，实行重大决策公示、听证和专家咨询制度，推进政府决策科学化、民主化。认真贯彻国务院《关于加强市县政府依法行政的决定》及我市实施依法行政纲要意见。健全行政复议和行政应诉及规范性文件审查备案制度。启动“十二五”规划前期工作。深化政务公开和政府信息公开，自觉接受新闻舆论和社会公众监督。

坚持为民、惠民、利民。加强社会管理，创新公共服务，推动公共财政向农村倾斜、向民生倾斜。重视解决涉及人民群众切身利益的问题，抓好市委、市政府10件23项为民办实事项目。依法加强信访工作，畅通民意表达渠道，继续完善领导接访群众制度。充分发挥“政风行风直通车”、“一线通”、“政风行风热线”、“新闻第一线”等平台作用，确保人民群众的合理诉求得到充分表达和妥善处理。针对当前部分中小企业经营困难的情况，在企业项目落地、生产经营、要素保障、市场开拓等方面改进服务，提高办事效率，形成有利于企业克服困难、转型升级和持续发展的环境。

坚持高效、务实、廉洁。启动第九轮行政审批制度改革，强化全程式网上审批运作，不断拓展并联审批和绿色通道服务范围，提高审批效率。全面推行规范行政自由裁量权工作。做好新一轮政府机构改革的相关工作。加强机关效能和绩效评估，转变机关工作作风，强化部门协同协作。加大督查督办力度，健全目标责任体系和考核制度。加强公务员队伍教育管理。落实行政问责制，坚决处理不作为、乱作为和执法不公行为。深化节约型机关建设，确保公用经费定额实现零增长，降低行政成本，勤俭办一切事业。认真实施《泉州市贯彻落实〈建立健全惩治和预防腐败体系2008-2012年工作规划〉工作方案》，落实党风廉政建设责任制。加大行政监察、审计监督力度，加强对工程建设、土地出让、征地拆迁、规划审批等领域的监管，深入开展商业贿赂和中介组织专项整治工作，建设廉洁政府。

各位代表，新的一年任务异常艰巨，也充满机遇和希望。让我们更加紧密地团结在以胡锦涛同志为总书记的党中央周围，高举中国特色社会主义伟大旗帜，以邓小平理论和“三个代表”重要思想为指导，全面贯彻落实科学发展观，在市委领导下，振奋精神，坚定信心，奋力发展，为全面提升海峡西岸经济区现代化工贸港口城市建设水平而努力奋斗！
